# Cancer cell death induced by the NAD antimetabolite Vacor discloses the antitumor potential of SARM1

**DOI:** 10.1002/1873-3468.70169

**Published:** 2025-09-16

**Authors:** Giuseppe Ranieri, Andrea Lapucci, Giuseppe Orsomando, Nadia Raffaelli, Alberto Chiarugi, Daniela Buonvicino

**Affiliations:** ^1^ Department of Health Sciences, Section of Clinical Pharmacology and Oncology University of Florence Italy; ^2^ Department of Clinical Sciences (DISCO), Section of Biochemistry Polytechnic University of Marche Ancona Italy; ^3^ Department of Agricultural, Food and Environmental Sciences Polytechnic University of Marche Ancona Italy

**Keywords:** cancer cells, NMNAT2, SARM1, Vacor

## Abstract

In a previous study, we showed that the NAD antimetabolite Vacor is metabolized by two enzymes implicated in the NAD salvage pathway—to Vacor mononucleotide (VMN) by nicotinamide phosphoribosyltransferase (NAMPT) and, in turn, to Vacor adenine dinucleotide (VAD) by nicotinamide mononucleotide adenylyltransferase 2 (NMNAT2)—leading to NAD depletion and antitumor activity. Recent findings in neurons show that VMN activates SARM1, a NAD glycohydrolase, triggering NAD depletion and degeneration. In this study, we report that altering NMNAT2 levels did not affect Vacor‐induced NAD depletion or cell death. In contrast, SARM1 expression alone was sufficient to induce Vacor sensitivity. Further, we report that cancer cells sense the abnormal expression of SARM1 and readily induce the expression of NMNAT2. Overall, the data underscore the antitumor potential of pharmacological approaches aimed at activating SARM1.

## Graphical abstract

Vacor, a compound converted into the toxic metabolite Vacor adenine dinucleotide (VAD) by the nicotinamide salvage pathway enzymes NAMPT and NMNAT2, exhibits antitumor activity by inducing rapid and complete NAD depletion. We report that Vacor toxicity is limited to cell lines expressing high levels of SARM1, a NAD glycohydrolase. These SARM1‐positive cell lines also show elevated expression of NMNAT2. Our data reveal SARM1 activation as a novel anticancer therapeutic strategy.

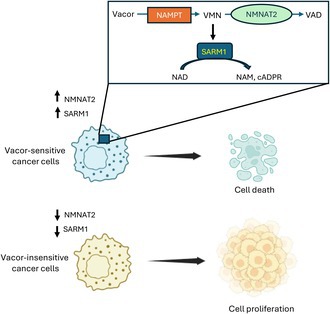

## Abbreviations

**NAD**, nicotinamide adenine dinucleotide

**NAM**, nicotinamide

**NAMPT**, nicotinamide phosphoribosyltransferase

**NMN**, nicotinamide mononucleotide

**NMNAT2**, nicotinamide mononucleotide adenylyltransferase2

**SARM1**, Sterile alpha and TIR motif‐containing protein 1

**VAD**, Vacor adenine dinucleotide

**VMN**, Vacor mononucleotide

The discovery of NAD as a substrate for multiple enzymes involved in regulating key metabolic pathways—such as differentiation, proliferation, and gene transcription—has brought the metabolome of the dinucleotide to the forefront of intense research [1, 2, 3]. This has led to the development of strategies aimed at depleting cellular NAD levels as innovative anticancer therapies [4, 5]. In this context, we found that the rodenticide Vacor is metabolized by two enzymes operating in the nicotinamide salvage pathway, a central route able to resynthesize NAD from nicotinamide. Specifically, we reported that Vacor is converted by nicotinamide phosphoribosyltransferase (NAMPT) into Vacor mononucleotide (VMN), which is further converted by nicotinamide mononucleotide adenyltransferase 2 (NMNAT2) into Vacor adenine dinucleotide (VAD), a NAD antimetabolite. This, in turn, prompts rapid and massive NAD depletion, exerting anticancer effects *in vitro* and in human melanoma and neuroblastoma xenografts [6].

We hypothesized that inhibition of NAD salvaging from nicotinamide (NAM) due to the engagement of NAMPT and NMNAT2 in Vacor metabolism could have been responsible for the massive reduction in NAD contents, although the literature suggests that NAMPT inhibition leads to a more gradual decrease in NAD levels compared to that caused by Vacor exposure. However, the lack of NMNAT inhibitors has prevented us from understanding how NAD depletion occurs when NAD resynthesis is completely blocked through the inhibition of both NAMPT and NMNAT. Among the various NAD‐consuming enzymes, we investigated the possible activation of PARP1, which can rapidly exhaust the intracellular NAD pool with kinetics similar to those observed after Vacor exposure [7, 8, 9]. However, inhibition of PARP1 did not prevent this NAD decline [6]. Another enzyme able to induce rapid intracellular NAD depletion is Sterile alpha and TIR motif‐containing protein 1 (SARM1), which is the central executioner of programmed axon degeneration and plays a key role in several models of neurodegenerative disorders [10, 11]. Originally known as a multidomain ubiquitous adaptor in Toll‐like receptor (TLR) signaling, SARM1 is now known to be a tightly regulated, multicatalytic NAD glycohydrolase that hydrolyzes NAD generating NAM, ADP ribose (ADPR), and cyclic ADPR (cADPR) [12] and also catalyzes intriguing pyridine base exchange reactions [13]. In solution, it is a native homo‐octamer of monomers composed of distinct domains, including an N‐terminal allosteric regulatory armadillo repeat (ARM) domain that prevents dimerization of catalytic TIR domains essential for SARM1 activation, thereby exerting a constitutive, autoinhibitory function. The SARM1 NAD‐consuming activity is mandatory for programmed axon degeneration and is finely tuned by the intracellular NAD/nicotinamide mononucleotide (NMN) ratio. While NAD inhibits SARM1 binding to the ARM domain, NMN binds to the same site but triggers SARM1 activation [14]. In a study in neurons, Coleman and colleagues report that the Vacor metabolite VMN activates SARM1 even more potently than NMN and prompts axonal NAD depletion and death [15]. Of note, while SARM1 is highly expressed in neurons, its expression levels and role in cancer cells remain unknown. It remains to be addressed, therefore, whether SARM1 activation contributes to Vacor cytotoxicity also in cancer cells.

In our previous study, we observed a correlation between NMNAT2 expression levels and Vacor sensitivity across different cell lines [6]. The selective toxicity of Vacor in cells expressing NMNAT2 and the identification of SARM1 as a target of VMN prompted us to investigate any possible correlation between these two enzymes. Of note, a key metabolic correlation between SARM1 and NMNAT2 activities in neuronal axons has been reported. Specifically, it has been demonstrated that NMNAT2 is an axonal survival factor by maintaining high NAD levels and therefore preventing SARM1 activation. In contrast, NMNAT2 depletion—leading to a rapid drop in axonal NAD levels and an increase in axonal NMN levels—triggers SARM1 activation, resulting in metabolic catastrophe and subsequent axon degeneration [15, 16, 17]. Whether a similar functional interaction between NMNAT2 and SARM1 exists in cancer cells remains unknown. On the one hand, SARM1 activation by Vacor in neurons has provided a valuable model for studying the enzyme's involvement in neurodegeneration. On the other hand, given the antitumor potential of Vacor‐like molecules, it is important to clarify whether SARM1 plays a role in cancer cells and to define the role of NMNAT2 in SARM1‐dependent Vacor‐induced cell death.

Based on these considerations, this study aimed to investigate the contribution of NMNAT2 and SARM1 to Vacor‐induced NAD depletion and cell death in cancer cell lines.

## Materials and methods

### Cell culture

HeLa (RRID: CVCL_0030), SH‐SY5Y (RRID: CVCL_0019), A375 (RRID: CVCL_0132), HEK (RRID: CVCL_M624), TT (RRID: CVCL_1774), CA77 (RRID: CVCL_8177), Neuro2a (RRID: CVCL_0470), A2780 (RRID: CVCL_0134), HEL (RRID: CVCL_0001), U87MG (RRID: CVCL_0022), Jurkat (RRID: CVCL_0065), PC3 (RRID: CVCL_0019), U251MG (RRID: CVCL_0021), LLC1 (RRID: CVCL_4358), and C26 (RRID: CVCL_XC68) cell lines were obtained from the American Type Culture Collection. Patient‐derived M26c (gender female) melanoma cells were obtained from human melanoma samples after approved protocols by the Ethics Committee, in accordance with the guidelines set by the Declaration of Helsinki [18]. All cell lines used in this study have been authenticated by short tandem repeat (STR) profiling within the past 3 years. Authentication was performed following the ANSI/ATCC ASN‐0002 standard. Genomic DNA was extracted, and STR profiles were compared against reference profiles and the Cellosaurus database to confirm identity and detect possible cross‐contamination. All cell lines were regularly tested and confirmed to be free of mycoplasma contamination. Cells were grown in Dulbecco's modified Eagle's medium (DMEM) containing 25 mm glucose and supplemented with 2 mm glutamine, 1 mm pyruvate, 10% fetal bovine serum, and antibiotics. Cultures were brought to 50–70% confluence before exposing them to the different compounds. Vacor (Greyhound Chromatography And Allied Chemicals Ltd, Birkenhead, UK) was dissolved in water with 4% of HCl 1 N. 5‐iodoisoquinoline (Merck, Milan, Italy) was dissolved in DMSO. All results are expressed as percentages of the control (untreated cells or vehicle) and each sample was normalized by the protein content.

### NAD and MTT measurement

NAD contents were quantified through enzymatic cycling as reported [19]. Briefly, to measure NAD contents, cells grown in a 48‐well plate were killed with 50 μL of 1 m HClO4 and then neutralized with an equal volume of 1 N KOH. After the addition of 50 μL of bicine (100 mm), 100 μL of the cell extract was then mixed with an equal volume of the bicine buffer containing ethanol, 3‐(4,5‐dimethylthiazol‐2‐yl)‐2,5‐diphenyltetrazolium, phenazine ethosulfate, and alcohol dehydrogenase. The mixture was kept at room temperature for 10 min, and then absorbance at 550 nm was measured (VICTOR3, PerkinElmer). Cell viability was assessed by 3‐(4,5‐dimethylthiazol‐2‐yl)‐2,5‐diphenyltetrazolium (MTT) assay, and absorbance at 550 nm was measured (VICTOR3, PerkinElmer) [20].

### Quantitative PCR

Total RNA was isolated using Trizol Reagent (Life Technologies, Monza, Italy). One μg of RNA was retrotranscribed using iScript (Bio‐Rad, Milan, Italy). RT‐PCR was performed as reported [21]. The following primers were used for the human: NMNAT1 forward 5′‐TCCCATCACCAACATGCACC‐3′, reverse 5′‐TGATGACCCGGTGATAGGCAG‐3′; NMNAT2 forward 5′‐GATTGGATCAGGGTGGACC‐3′, reverse 5′‐TCCGATCACAGGTGTCATGG‐3′; SARM1 forward 5′‐GCAGTAGCGGTGTTGGCGACTAAC‐3′, reverse 5′‐TTAGAGTCGAGCAACGGCACGAGG‐3′; 18S forward 5′‐CGGCTACCACATCCAAGGAA‐3′, reverse 5′‐GCTGGAATTACCGCGGCT‐3′. The following primers were used for the mouse: NMNAT1 forward 5′‐AACAGATGTGCCCAAGGTG‐3′, reverse 5′‐CTCCACAGCACATCGGACTC‐3′; NMNAT2 forward 5′‐GGGGACTTTGGGATCGTCGTGG‐3′, reverse 5′‐GGTATCCGCTAGCCCGAGGC‐3′; SARM1 forward 5′‐CGAGGTCGAGCATTCTGGCACATTG‐3′, reverse 5′‐CTCTGCGCACAGGTAGAATGCTCC‐3′; 18S forward 5′‐AAAACCAACCCGGTGAGCTCCCTC‐3′, reverse 5′‐CTCAGGCTCCCTCTCCGGAATCG‐3′. Primers were purchased from Integrated DNA Technologies (Coralville, IA, USA).

### NMNAT2 and SARM1 silencing and overexpression

Nine thousand cells were subcultured in 48‐well plates and then incubated with 50 nm NMNAT2 or SARM1 siRNA. After 24 h, cells were exposed or not to Vacor 100 μm, and after different times, the NAD content and MTT reduction were measured, or RNA was extracted. Validated siRNA for human NMNAT2 (hs.Ri.NMNAT2.13.2) and SARM1 (hs.Ri.SARM1.13.2) were used (IDT, Coralville, IA, USA, Tema Ricerca).

Overexpression experiments of human NMNAT2 and SARM1 were carried out in HeLa cells. Briefly, the coding region of NMNAT2 was amplified using specific primers, with the reverse primer including a Flag tag at the 3' end. The amplified product was then cloned into the pCDNA3 vector (Life Technologies), generating the pCDNA3‐hNMNAT2‐Flag construct. Similarly, the coding region of SARM1 was amplified with specific primers and cloned into the same vector to generate the pCDNA3‐hSARM1 construct. Both plasmids were verified by nanopore sequencing (Oxford Nanopore Technologies, Oxford, UK) to confirm the accuracy of the nucleotide sequences. HeLa cells were seeded in 48‐well plates at a density of 1 × 10^5^ cells per well. After 24 h, cells were transfected with 50 ng of either pCDNA3‐hNMNAT2‐Flag or pCDNA3‐hSARM1 plasmids using jetPRIME^®^ (Polyplus, Illkirch, France), following the manufacturer's instructions. pCDNA3 empty vector was used as a negative control.

### NMNAT enzymatic assay

Cytosol, mitochondria, and nuclei were isolated from HeLa cells using a glass/glass homogenizer in 500 μL of extraction buffer (25 mm Tris/HCl pH 8, 150 mm NaCl, 0.5% Triton‐X). Briefly, supernatants were first centrifuged at 600 ***g*** to obtain the nuclear fraction, and then at 7000 ***g*** to obtain the mitochondrial pellet and cytosol. For the enzymatic assay, the cytosolic fraction was incubated for 30 min at 37 °C in a reaction mixture containing 50 mm Tris/HCl pH 8, 200 mm NaCl, 1 mm MgCl2, 1 mm NMN, and 1 mm ATP. NAD formation was evaluated using the abovementioned enzymatic cycling procedure [19].

### Western blot

Cell lines were dissolved in 1% SDS. BCA (bicinchoninic acid) Protein Assay was used to quantify the total protein content. Lysates (20 μg/lane of protein) were resolved by electrophoresis on a 4 to 20% SDS‐polyacrylamide gel (Bio‐Rad Laboratories, Hercules, CA, USA) and transferred onto nitrocellulose membranes. After blocking, the blots were incubated overnight at 4 °C with human SARM1 (D2M5I from Cell Signaling, Danvers, MA, USA), mouse SARM1 (A23440 from Ab Clonal, Düsseldorf, Germany), human NMNAT1 (ab118270 from Abcam, Cambridge, London, UK), or human NMNAT2 (SC‐515206 from Santa Cruz Biotechnology, Heidelberg, Germany) in TBS‐T containing 5% bovine serum albumin. Tubulin (PAS‐29444 from Invitrogen, Waltham, MA, USA) or GAPDH (G8795, Merck) was used as a loading control. Immunodetection was performed with HRP‐conjugated secondary antibodies (diluted 1 : 2000 anti‐mouse) (Amersham Biosciences, Little Chalfont, UK) in TBS‐T containing 5% nonfat dry milk. After washing, the membranes were detected using chemiluminescence (ECL plus; Euroclone, Padova, Italy). Quantity One analysis software was used for quantitative analysis (Bio‐Rad). Results are presented as the mean standard error of the mean (SEM) of different gels and expressed as Arbitrary Units, which depicts the ratio between levels of target protein expression and tubulin normalized to basal levels [22]. To evaluate the basal NMNAT2 contents in different cell lines, additional antibodies were tested: human NMNAT2 (WH 0023057M1 from Merck), mouse NMNAT2 (A13833 from ABclonal, Busseldorf, Germany), human/mouse NMNAT2 (ab56980 from Abcam, Milan, Italy).

### Determination of vacor metabolites in HeLa cells

Vacor metabolites were determined as described [6] with minor modifications. Briefly, pellets of HeLa cells were resuspended in 0.4 m HClO_4_. After 5 min on ice, samples were centrifuged at 16 000 ***g*** for 5 min; supernatants were neutralized with 1 m K_2_CO_3_ and centrifuged as above. Neutralized supernatants corresponding to about 1 mg of proteins were injected into a Supelcosil LC‐18‐S column equilibrated with 33% buffer A (100 mm potassium phosphate buffer, pH 6.0, 8.0 mm tetrabutylammonium sulfate) and 67% buffer B (buffer A containing 30% methanol). Elution conditions were as follows: 16 min 67% buffer B, up to 100% buffer B in 1 minute, hold at 100% buffer B for 18 min, down to 67% buffer B in 5 min, hold at 67% buffer B for 5 min. The flow rate was maintained at 2.0 mL·min^−1^, and the column holder temperature was fixed at 40 °C.

### Statistical analysis

Data are presented as the mean values ± SEM. All differences among groups were performed using ANOVA followed by Tukey's *W*‐test or Student's *t*‐test. Levels of significance were *P* < 0.05 (*), *P* < 0.01 (**), and *P* < 0.001 (***). Statistical analyses were carried out using GraphPad Prism (version 7).

## Results

### Effects of NMNAT2 overexpression or silencing in Vacor‐insensitive or Vacor‐sensitive cancer cell lines

**Fig. 1:**
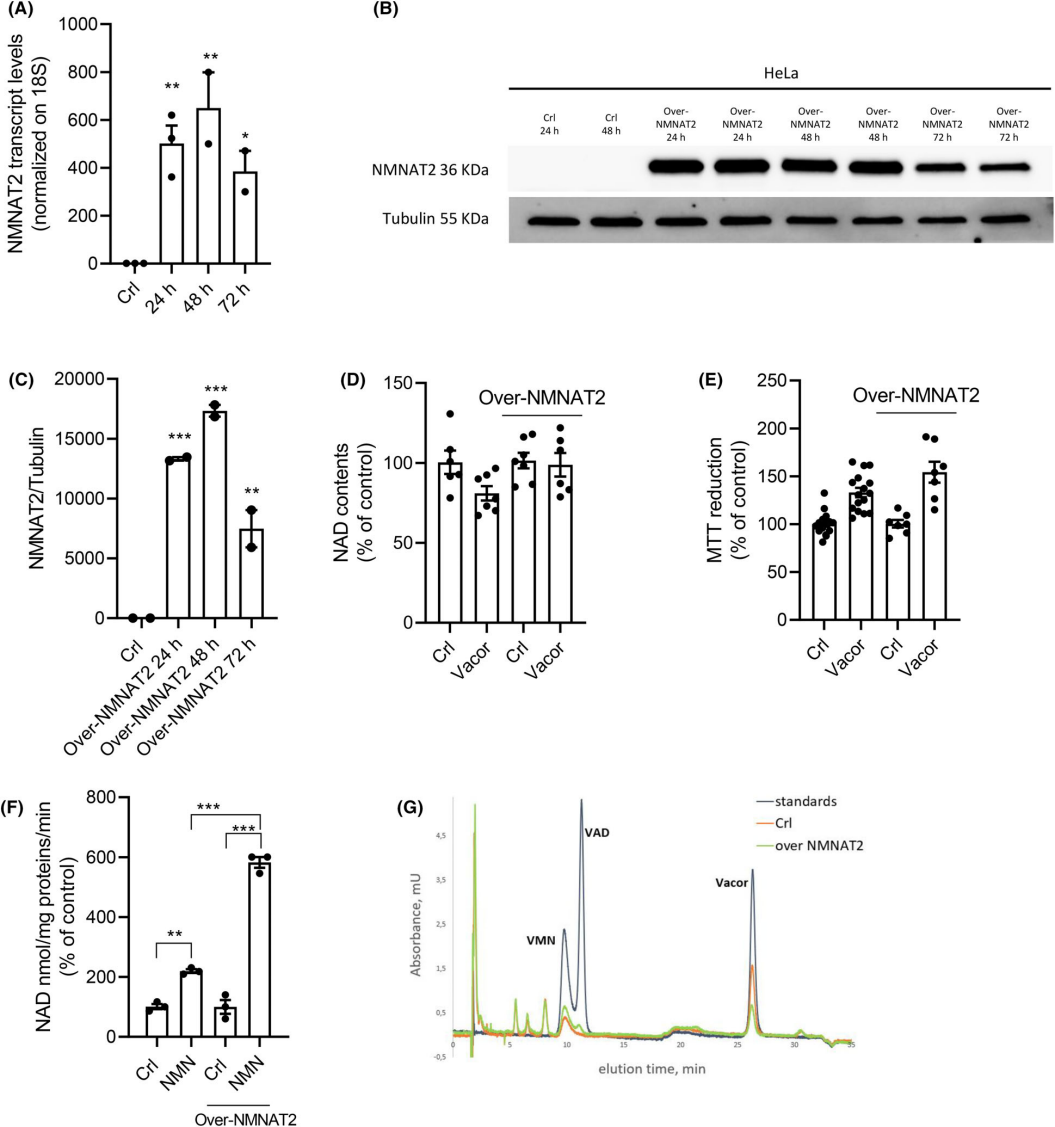
NMNAT2 overexpression does not induce sensitivity to Vacor. Effects of NMNAT2 overexpression on its transcript (A) and protein (B, C) levels in HeLa cells after 24, 48, and 72 h. Effects of 48‐h NMNAT2 overexpression on intracellular NAD contents (D) and MTT reduction (E) in HeLa cells after 24‐h Vacor exposure. Effects of 48‐h NMNAT2 overexpression on NAD production of HeLa cellular extracts incubated with NMN (1 mm) for 15 min (100% equal 81 ± 2.7 nmol/min/mg protein) (F). HPLC profiles at 340 nm of HeLa cells overexpressing or not NMNAT2 after Vacor exposure (100 μm/6 h) (G). In (A), (C), and (D), each column represents the mean ± SEM of two experiments. In (E) and (F), each column represents the mean ± SEM of three experiments. *P < 0.05, **P < 0.01, and ***P < 0.001 versus Crl. ANOVA and Tukey's *post hoc* test were used.

**Fig. 2:**
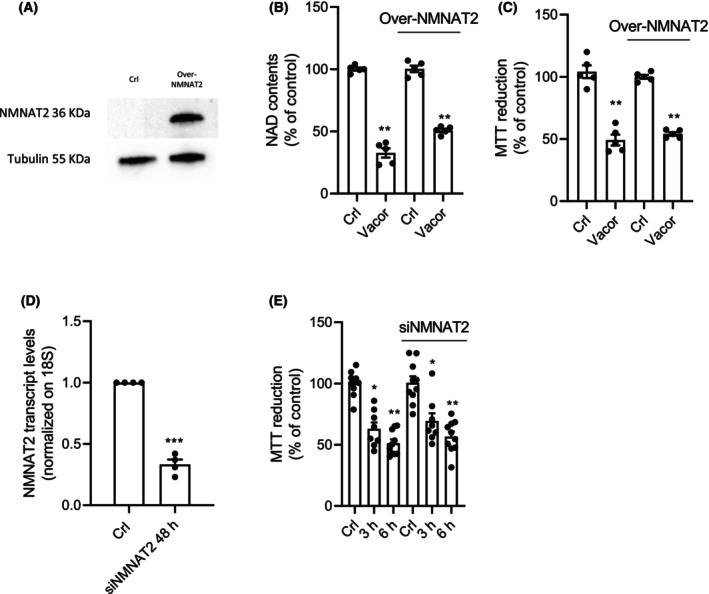
NMNAT2 overexpression or silencing does not increase or prevent Vacor toxicity. Effects of 48‐h NMNAT2 overexpression on its protein levels in SH‐SY5Y cells (A). Effects of 48‐h NMNAT2 overexpression on intracellular NAD contents (B) and MTT reduction (C) in SH‐SY5Y cells after 4‐h Vacor exposure). Effects of 48‐h NMNAT2 silencing on its transcript levels in SH‐SY5Y cells (D). Effects of 48‐h NMNAT2 silencing on MTT reduction in SH‐SY5Y cells exposed to Vacor the last 3 and 6 h (E). In (B) and (C), each column represents the mean ± SEM of two experiments. In (D) each column represents the mean ± SEM of four experiments. In (E) each column represents the mean ± SEM of three experiments. *P < 0.05, **P < 0.01, and ***P < 0.001 versus Crl. Student's *t*‐test was used in (D). ANOVA and Tukey's *post hoc* test were used in (B).

To assess the role of NMNAT2 in Vacor cytotoxicity, we tested first the effects of NMNAT2 overexpression in Vacor‐insensitive cell lines. HeLa cells are insensitive to Vacor and express very low NMNAT2 levels [19], so we selected this cell line to investigate the impact of NMNAT2 overexpression under Vacor exposure. Despite high NMNAT2 transcript (Fig. 1A) and protein (Fig. 1B,C) levels following plasmid transfection, we found that NMNAT2 overexpression did not sensitize HeLa cells to Vacor (Fig. 1D,E). To confirm that the overexpressed NMNAT2 protein was functional, we performed an enzymatic assay on nuclear‐free cell extracts from transfected and nontransfected cells. We found that the conversion of the substrate NMN to NAD was approximately three times higher in NMNAT2‐overexpressing HeLa cells (Fig. 1F). Consistent with this, only HeLa cells overexpressing NMNAT2 were able to synthesize VAD from Vacor (Fig. 1G), providing further evidence of NMNAT2's enzymatic activity. We then moved to analyze modulation of NMNAT2 expression in the Vacor‐sensitive SH‐SY5Y neuroblastoma cell lines. Surprisingly, at variance with the putative role of NMNAT2 in mediating Vacor's cytotoxicity, we found that its overexpression in SH‐SY5Y cells (Fig. 2A) neither exacerbated NAD depletion nor accelerated cell death (Fig. 2B,C). Accordingly, even silencing of NMNAT2 (Fig. 2D) did not affect Vacor toxicity in SH‐SY5Y cultured cells (Fig. 2E).

Taken together, these data indicate that NMNAT2 is not required for the toxic effects of Vacor.

### SARM1 expression levels and Vacor cytotoxicity in cancer cell lines

**Fig. 3:**
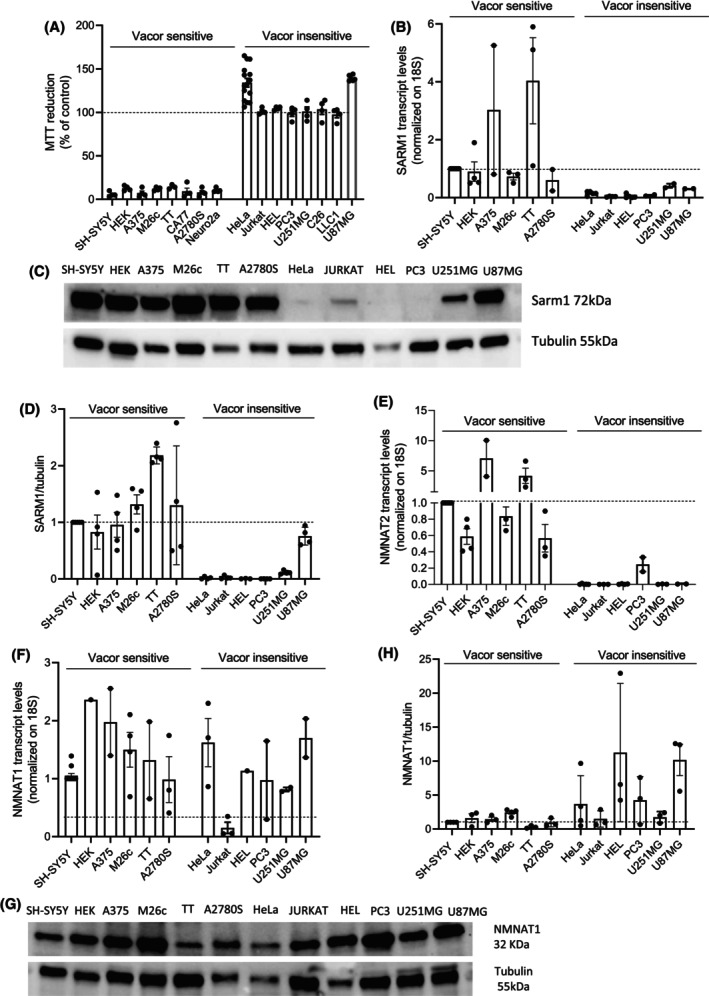
SARM1 and NMNAT2 are coexpressed and their expression correlates with the sensitivity to Vacor. MTT reduction in several cancer cell lines exposed to Vacor 100 μm for 24 h (A). Transcript (B) and protein (C, D) levels of SARM1 in Vacor‐sensitive and Vacor‐insensitive cells are shown. In (E) transcript levels of NMNAT2 in Vacor‐sensitive and Vacor‐insensitive cell lines are shown. Transcript (F) and protein (G, H) levels of NMNAT1 in Vacor‐sensitive and Vacor‐insensitive cells are shown. In (A, B, E), and (F), each column represents the mean ± SEM of two experiments. In (D), each column represents the mean ± SEM of four experiments. In (H), each column represents the mean ± SEM of three experiments.

We next assessed the expression levels of SARM1 in various Vacor‐sensitive and ‐insensitive cancer cell lines. We found that, in addition to the previously identified SH‐SY5Y (neuroblastoma), M26C and A375 (melanoma) cell lines, Vacor also induced rapid cell death in HEK (embryonic kidney), HTT and CA77 (both from thyroid medullary carcinoma), Neuro2a (neuroblastoma), and A2780 (ovarian carcinoma) cell lines. Conversely, we found that, similarly to HeLa cells, HEL (erythroleukemia), U87MG and U251 (glioblastoma), Jurkat (T‐cell leukemia), PC3 (prostate cancer), LLC1 (Lewis lung carcinoma), and C26 (colon adenocarcinoma) cells were insensitive to Vacor (Fig. 3A). Interestingly, we observed that SARM1 transcript levels were higher in Vacor‐sensitive than in insensitive cell lines (Fig. 3B). This correlation also occurred for SARM1 protein levels, with the exception of the glioblastoma cells U87MG and U251 (Fig. 3C,D). Indeed, the latter, in spite of high SARM1 expression, were Vacor insensitive (Fig. 3A). Surprisingly, we also found that U87MG and U251 cells undergo NAD depletion when exposed to Vacor (Fig. S1), further highlighting the unique resistance of glioblastoma to cytotoxic agents.

**Fig. 4:**
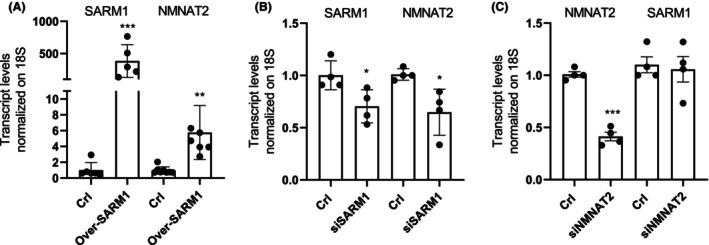
Silencing or overexpression of SARM1 regulates NMNAT2 expression. Effects of 48‐h SARM1 overexpression on its and NMNAT2 transcript levels in HeLa cells (A). Effects of 48‐h SARM1 silencing on its and NMNAT2 transcript levels in SH‐SY5Y cells (B). Effects of 48‐h NMNAT2 silencing on its and SARM1 transcript levels in SH‐SY5Y cells (C). *P < 0.05 and ***P < 0.001 versus Crl. ANOVA and Tukey's *post hoc* test were used. Each column represents the mean ± SEM of two experiments.

Consistent with our previous findings, we observed that Vacor‐sensitive cell lines also exhibit high expression levels of NMNAT2 (Fig. 3E), highlighting a correlation between the transcripts of *SARM1* and *NMNAT2* genes. Unfortunately, none of the three NMNAT2 antibodies we used (see Methods) was able to detect the enzyme's basal expression levels (Fig. 2A and S2). This prevented us from making a direct correlation between SARM1 and NMNAT2 protein levels in the different cell lines. Of note, the correlation between SARM1 and NMNAT2 expression levels appears specific, as no correlation was observed with the isoform NMNAT1 (Fig. 3F–H and Fig. S3). To further explore the relationship between these two proteins, we also assessed the effect of modulating SARM1 expression on NMNAT2 levels. Remarkably, we found that overexpression of SARM1 led to an increase in NMNAT2 transcript levels in HeLa cells (Fig. 4A), whereas SARM1 silencing resulted in reduced NMNAT2 expression in SH‐SY5Y cells (Fig. 4B). However, NMNAT2 silencing had no effects on SARM1 expression in SH‐SY5Y cells (Fig. 4C).

Collectively, these data indicate a correlation between SARM1 levels and Vacor sensitivity, except in glioblastoma cell lines. Additionally, the findings suggest a relationship between the expression levels of SARM1 and NMNAT2, indicating that changes in SARM1 expression influence NMNAT2 levels.

### Effects of SARM1 silencing or overexpression in Vacor‐sensitive or ‐insensitive cancer cell lines

**Fig. 5:**
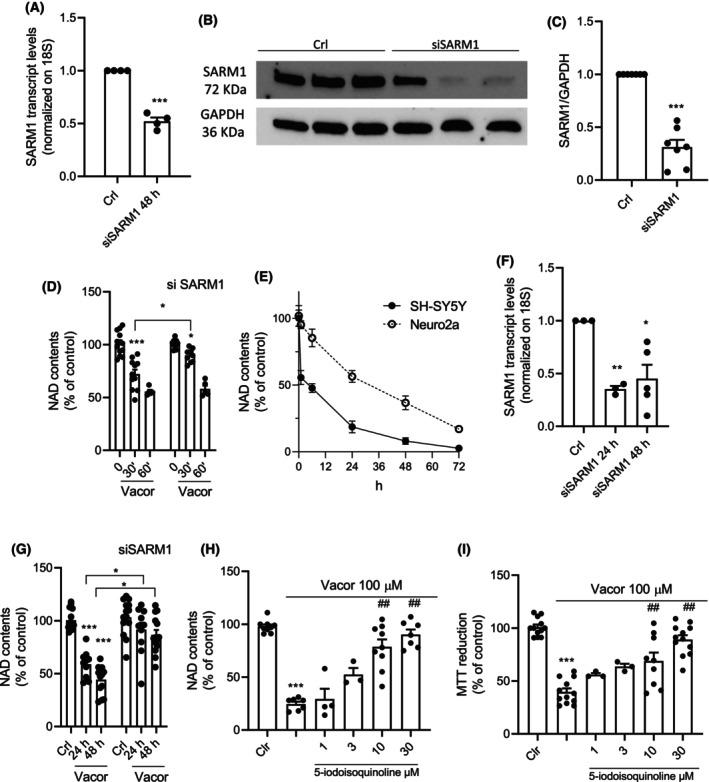
SARM1 silencing or pharmacological inhibition counteracts Vacor toxicity. Effects of 48‐h SARM1 silencing on its transcript (A) and protein (B and C) levels in SH‐SY5Y cells. Effects of 48‐h SARM1 silencing on intracellular NAD contents in SH‐SY5Y cells exposed to Vacor (100 μm) the last 30 and 60 min (D). Time course of intracellular NAD contents in SH‐SY5Y and Neuro2a cells exposed to Vacor (100 μm) (E). Effects of 24 and 48‐h SARM1 silencing on its transcript levels in Neuro2a cells (F). Effects of 24‐h SARM1 silencing on intracellular NAD contents in Neuro2a cells exposed to Vacor (100 μm) for 24 h from the time of silencing after (G). Effects of different concentrations of 5‐iodoisoquinoline (1–30 μm) on intracellular NAD contents (H) and MTT reduction (I) in SH‐SY5Y cells exposed to Vacor for 24 h. *P < 0.05, **P < 0.01, and ***P < 0.001 versus Crl; ^##^ *P* < 0.01 versus Vacor. ANOVA and Tukey's *post hoc* test were used. In (A, C) and (G), each column represents the mean ± SEM of four experiments. In (A, C), and (G), each column represents the mean ± SEM of foueriments. In (D–F, H, I), each column represents the mean ± SEM of three experiments.

To further investigate how SARM1 expression correlates with Vacor‐dependent cytotoxicity, we evaluated the effects of SARM1 silencing (Fig. 5A–C) in Vacor‐sensitive SH‐SY5Y cells. Interestingly, silencing of SARM1 reduced NAD drop caused by Vacor exposure only at early time points (30 min) (Fig. 5D). However, when we silenced SARM1 in the Neuro2a neuroblastoma cell line, which undergoes lower NAD depletion kinetics compared to SH‐SY5Y cells after Vacor exposure (Fig. 5E), we found that silencing (Fig. 5F) completely prevented Vacor‐dependent NAD depletion (Fig. 5G). In keeping with this, the SARM1 inhibitor 5‐iodoisoquinoline dose‐dependently counteracted both NAD depletion and cytotoxicity in SH‐SY5Y cell cultures exposed to Vacor (Fig. 5H,I).

**Fig. 6:**
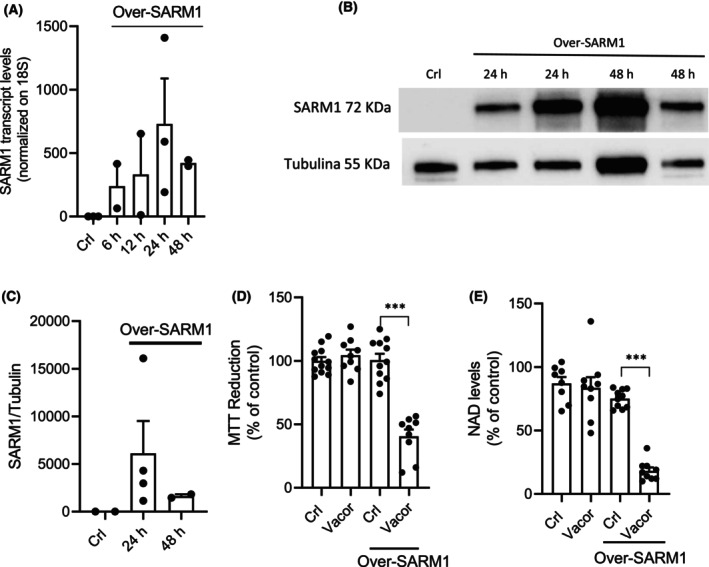
SARM1 overexpression sensitizes to Vacor toxicity. Effects of SARM1 overexpression on its transcript (A) and protein (B, C) levels in HeLa cells after plasmid transfection. Effects of 24‐h SARM1 overexpression on intracellular NAD contents (D) and MTT reduction (E) in HeLa cells exposed to Vacor (100 μm). ***P < 0.001 versus Crl. ANOVA and Tukey's *post hoc* test were used. In (A), each column represents the mean ± SEM of two experiments. In (C–E), each column represents the mean ± SEM of four experiments.

Next, to also investigate the impact of SARM1 activation in Vacor‐insensitive cell lines, we explored the effects of SARM1 overexpression in HeLa cells. After transfection, we observed a 500‐fold increase in SARM1 transcripts after 6 h (Fig. 6A) and in protein levels at 24 and 48 h (Fig. 6B,C). Notably, SARM1 overexpression sensitized HeLa cells to Vacor‐dependent NAD depletion and cell death (Fig. 6D,E).

Overall, data show a key role of SARM1 in Vacor‐induced NAD depletion and death in cancer cell lines.

## Discussion

Chemicals able to target tumor cell metabolism represent key tools for cancer therapy. It is well known that NAD availability is crucial to sustain the high bioenergetic requirement of neoplasms, and different pharmacological approaches have been developed to counteract NAD resynthesis within cancer cells [23]. In this scenario, we previously identified Vacor as a tumor antimetabolite toxified to VAD via NAMPT and NMNAT2, and able to prompt rapid and complete NAD depletion. Because of the selective toxicity of Vacor in cancer cells expressing NMNAT2, we originally hypothesized this enzyme is a prerequisite to exert such an antitumor activity [6].

Subsequent work by Coleman and his group, however, focused on the neurotoxic effects of Vacor and reported that its ability to prompt NAD depletion and neuronal death is due to massive SARM1 activation by VMN, a structural analog of the endogenous SARM1 activator NMN [15, 24]. These findings prompted us to investigate whether NMNAT2 and/or SARM1 activity underpins the anticancer effects in Vacor.

We now report, in contrast with our prior hypothesis, that silencing of NMNAT2 in Vacor‐sensitive cancer cell lines neither reduces NAD depletion nor protects from the compound's cytotoxic effect. Consistently, NMNAT2 overexpression does not sensitize cancer cells to Vacor toxicity. Collectively, these findings suggest that NMNAT2 does not contribute to Vacor‐dependent NAD loss and anticancer activity. It is worth noting, however, that NMNAT2 overexpressing cells remain viable in spite of the accumulation of intracellular VAD. Again, this is at odds with our previous finding that exposure of cancer cells to exogenous VAD is sufficient to trigger cell death. In an attempt to reconcile these findings, we emphasize that while death by exogenous VAD occurs with delayed kinetics of about 96 h, Vacor prompts cell demise rapidly, within 8–12 h [6]. Hence, we infer that on the one hand exogenous VAD exerts cytotoxic events unrelated to those activated by Vacor, and on the other that Vacor‐dependent VAD accumulation *per se* is not sufficient to trigger cell death. Alternatively, we speculate that resistance to intracellular VAD accumulation occurs because it takes place in cells transfected with NMNAT2 but lacking endogenous SARM1 expression. Indeed, in light of the ability of SARM1 to directly synthesize VAD from Vacor via base exchange with NAD [13], the possibility exists that, in contrast to Vacor‐sensitive cell lines that express both NMNAT2 and SARM1, in cancer cells exclusively overexpressing NMNAT2 the amount of VAD produced is insufficient to induce cell death.

It is well known that SARM1 is abundantly expressed in neurons, and recent studies report its expression also in immune cells such as macrophages and T cells [25, 26]. For the first time, our study shows that SARM1 expression varies among different cancer cell lines. Remarkably, we also report that the sensitivity of cancer cells to Vacor toxicity is restricted to those lines expressing high levels of SARM1, thereby indicating that Coleman's claim that SARM1 is causative of Vacor neurotoxicity can also be applied to cancer cells. An apparent, notable exception appears to be glioblastoma. We found, indeed, that two glioblastoma cell lines express relatively high levels of SARM1 but do not display Vacor sensitivity. Resistance to the chemical might be ascribed to the ability of glioblastoma cells to undergo only partial (50%) NAD depletion after prolonged Vacor exposure, suggesting sub‐lethal SARM1 activation levels in glioblastoma. Remarkably, this neoplasm, one of the most difficult to treat, might possess a unique regulation of NAD homeostasis/SARM1 activity that needs to be investigated in future studies.

A key finding of the present study is that cancer cell lines expressing SARM1 also exhibit high levels of NMNAT2. Data suggest that the correlation between NMNAT2 and SARM1 expression levels observed in mammalian axons [16] also occurs in cancer cells. Of note, this correlation appears to be specific for NMNAT2, given that we did not find a relationship between NMNAT1 and SARM1 expression levels in cancer cells. Curiously, high levels of NMNAT2 are associated with increased metabolic activity in cancer cells and correlate with growth rate and prognosis of malignant tumors [27]. Although the role of elevated SARM1 levels in this context remains unclear, it has been reported that high NMNAT2 levels prevent SARM1 activation in neurons [16]. Accordingly, constitutively low NMNAT2 expression associates with sublethal SARM1 activation which, in turn, predisposes to human axonal disorders [28]. We reason that the correlation between the expression levels of these two enzymes has a similar cytoprotective/proliferating significance in cancer. Indeed, cells expressing high levels of SARM1 would be at risk of NAD depletion and would benefit from concomitant high expression of NMNAT2 that transforms the SARM1 activator NMN into the SARM1 inhibitor and bioenergetic cofactor NAD. In other words, NMNAT2 expression could negatively regulate SARM1 activation via modulation of NAD metabolism, thereby creating a feedback loop also in cancer cells. In keeping with this interpretation, our experiments silencing or overexpressing SARM1 indicate that the NADase levels regulate those of NMNAT2. Interestingly, this mechanism appears to be unidirectional, as only SARM1 drives NMNAT2 expression and not *vice versa*. Although further studies are needed to identify the molecular mechanisms underlying this transcriptional relationship, our data suggest that maintaining the NMNAT2/SARM1 balance is crucial for cancer cells homeostasis and tumor biology. However, this correlation between SARM1 and NMNAT2 may also appear paradoxical. Indeed, SARM1 is primarily known to localize to mitochondria, and the regulation of the cytosolic isoform NMNAT2—rather than the mitochondrial isoform NMNAT3—to maintain NAD levels following SARM1 upregulation may seem inconsistent. This apparent contradiction can be explained by considering, on the one hand, that SARM1 is also present in the cytosol [29], and on the other hand, that the identification of a mitochondrial NAD transporter [30] suggests an additional mechanism—besides NMNAT3 [31]—for maintaining mitochondrial NAD levels. We can also speculate that the increase in NMNAT2 expression following SARM1 upregulation may contribute to a general rise in cellular NAD levels, although not specifically within mitochondria. From an evolutionary perspective, this effect might have been selectively preserved to enhance the cellular response to SARM1 activation and thereby prevent cell death. Furthermore, the observation that SARM1 can still drive degeneration even without a mitochondrial targeting sequence [32] highlights that its activity is not exclusively related to mitochondria but suggests a dynamic role between cellular compartments.

Smoldering SARM1 activation levels might indeed influence both cancer cell survival and proliferation, as well as correlate with the development and prognosis of malignant tumors. We claim that understanding the correlation between NMNAT2 and SARM1 activity could have important therapeutic implications and represent a biomarker of tumor aggressiveness. Finally, another implication of our findings is that antitumoral Vacor‐like compounds may exhibit selective toxicity toward tumors expressing SARM1, which could serve as a predictive biomarker for therapeutic sensitivity. In this regard, evaluating SARM1 transcript and protein levels in biopsies might represent a key step to corroborate our data *in vitro*.

Overall, our study underscores the pivotal role of SARM1 in Vacor‐induced cytotoxicity in cancer cells, as well as its potential as an antineoplastic agent to design innovative avenues for cancer therapy.

## Conflict of interest

The authors declare no conflict of interest.

## Author contributions

GR, AL, NR, GO, and DB: Data curation, Formal analysis, Investigation, Methodology; DB, NR, GO, and AC: Conceptualization, Visualization. AC and DB: Project administration, Supervision; AC: Funding acquisition; DB: writing – original draft.

## Supporting information


**Fig. S1.** Effects of Vacor on intracellular NAD contents in glioblastoma cell lines.

**Fig. S2.** NMNAT2 protein expression levels on basal condition in several Vacor‐sensitive and ‐insensitice cell lines.

**Fig. S3.** SARM1, NMNAT2, and NMNAT1 expression in Vacor‐sensitive and ‐insensitive mouse cell lines.

## Data Availability

The data that support the findings of this study are available upon request from the corresponding author.
