# Supplementary material for: Cancer cell death induced by the NAD antimetabolite Vacor discloses the antitumor potential of SARM1
Source: FEBS Lett. 2025 Sep 16;599(21):3150–62. doi: 10.1002/1873-3468.70169 (PMC12599613; doi:10.1002/1873-3468.70169)
Supplement: Supplementary file 1 — Fig. S1. Effects of Vacor on intracellular NAD contents in glioblastoma cell lines. [file FEB2-599-3150-s003.pdf]

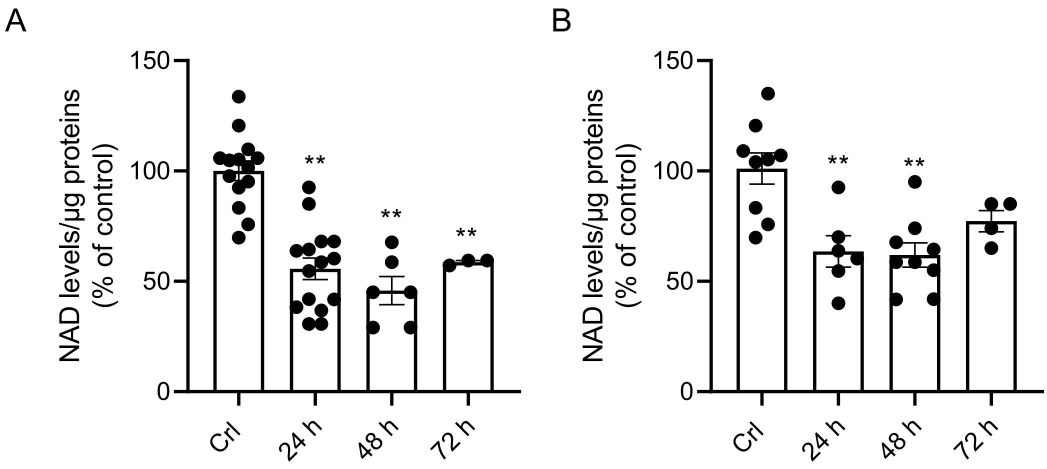

Supplementary Figure 1. Effects of Vacor on intracellular NAD contents in glioblastoma cell lines. Effects of Vacor 100µM on NAD levels in glioblastoma U87MG (A) and U251MG (B) cells. ∗∗$p < 0.01$ versus Crl. ANOVA and Tukey's post hoc test were used. Each column represents the mean ± SEM of three experiments.

Figure S1
