# Supplementary material for: Cancer cell death induced by the NAD antimetabolite Vacor discloses the antitumor potential of SARM1
Source: FEBS Lett. 2025 Sep 16;599(21):3150–62. doi: 10.1002/1873-3468.70169 (PMC12599613; doi:10.1002/1873-3468.70169)
Supplement: Supplementary file 2 — Fig. S2. NMNAT2 protein expression levels on basal condition in several Vacor‐sensitive and ‐insensitice cell lines. [file FEB2-599-3150-s001.pdf]

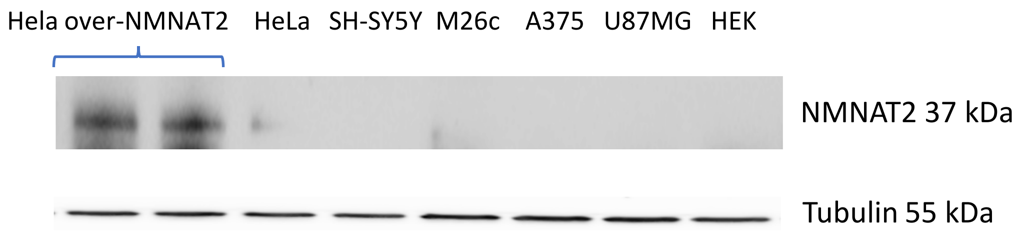

Supplementary Figure 2. **NMNAT2 protein expression levels on basal condition in several Vacor-sensitive and –insensitice cell lines.** Protein levels of NMNAT2 after 48h NMNAT2 overexpression (over-NMNAT2) in HeLa cells and in basal condition in several Vacor-sensitive (SH-SY5Y, M26c, A375, HEK) and –insensitive (U87MG and HeLa) cell lines.

Figure S2
