# Supplementary material for: Cancer cell death induced by the NAD antimetabolite Vacor discloses the antitumor potential of SARM1
Source: FEBS Lett. 2025 Sep 16;599(21):3150–62. doi: 10.1002/1873-3468.70169 (PMC12599613; doi:10.1002/1873-3468.70169)
Supplement: Supplementary file 3 — Fig. S3. SARM1, NMNAT2, and NMNAT1 expression in Vacor‐sensitive and ‐insensitive mouse cell lines. [file FEB2-599-3150-s002.pdf]

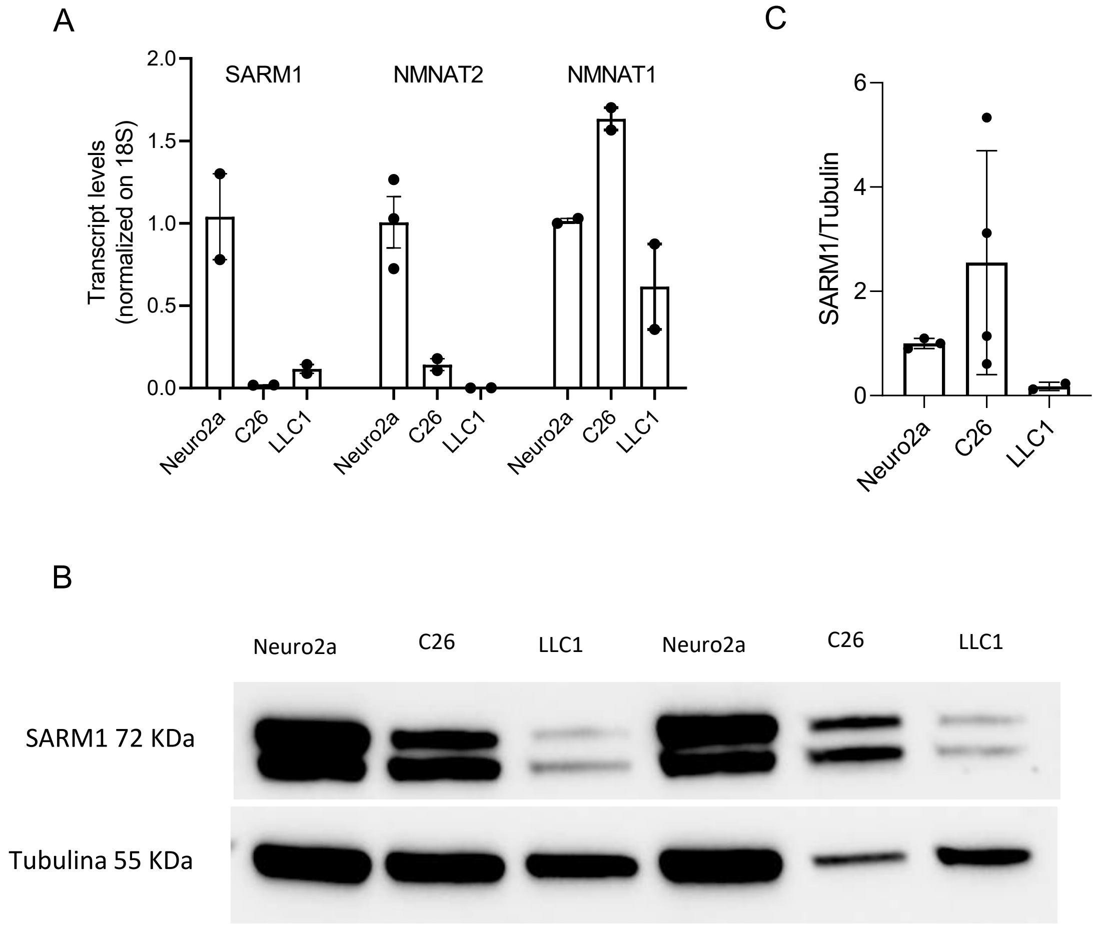

Supplementary Figure 3. SARM1, NMNAT2 and NMNAT1 expression in Vacor-sensitive and -insensitive mouse cell lines. Transcript levels of SARM1, NMNAT2 and NMNAT1 (A) and protein levels of SARM1 (B and C) in Vacor-sensitive (Neuro2a) and Vacor-insensitive (C26 and LLC1) cells. Each column represents the mean ± SEM of two experiments.

Figure S3
